# Customized Color Settings of Digitally Assisted Vitreoretinal Surgery to Enable Use of Lower Dye Concentrations During Macular Surgery

**DOI:** 10.3389/fmed.2021.810070

**Published:** 2022-01-24

**Authors:** Su Jin Park, Jae Rock Do, Jae Pil Shin, Dong Ho Park

**Affiliations:** ^1^Department of Ophthalmology, School of Medicine, Kyungpook National University, Kyungpook National University Hospital, Daegu, South Korea; ^2^Kyungpook National University Bio-Medical Research Institute, Daegu, South Korea; ^3^Kyungpook National University Cell and Matrix Research Institute, Daegu, South Korea

**Keywords:** vitrectomy, macular surgery, epiretinal membrane (ERM), ILM peeling, 25 gauge pars plana vitrectomy

## Abstract

**Purpose:**

This study evaluated the color contrast ratio (CCR) of the internal limiting membrane (ILM) using different color settings of digitally assisted vitreoretinal surgery (DAVS) with different indocyanine green (ICG) concentrations.s

**Methods:**

This is a prospective comparative observational study. Consecutive patients that underwent 25G vitrectomy and ILM peeling using a standard operating microscope (SOM) (25 eyes), DAVS Ver. 1.1 (12 eyes), or DAVS Ver. 1.3 (13 eyes) were enrolled. The SOM and DAVS Ver. 1.1 groups used 0.075% ICG, and the DAVS Ver. 1.3 group used 0.025% ICG. In DAVS Ver. 1.1, macular CCR was compared between four different presets in the red, green, and blue channels: Default (Red (R) 100%, Green (G) 100%, and Blue (B) 100%); Preset 1 (R 20%, G 100%, B 100%); Preset 2 (R 80%, G 80%, B 100%), and Preset 3 (R 85%, G 100%, B 90%). In DAVS Ver. 1.3, macular CCR was evaluated using two different customized settings that modified the hue and saturation: Customized Setting 1 (R 86, G 100, B 100%, Hue +2°, Saturation 90%, Gamma 1.2) and Customized Setting 2 (R 90, G 100, B 100%, Hue +20°, Saturation 100%, Gamma 0.9). All patients underwent ophthalmologic examinations including BCVA at baseline and at 12 months.

**Results:**

In DAVS Ver. 1.1, macular CCR was highest in Preset 3 (*P* < 0.01). The CCR of Customized Setting 2 of DAVS Ver. 1.3 using 0.025% ICG did not differ from that of Preset 3 in DAVS Ver. 1.1 using 0.075% ICG. Furthermore, there was no significant difference in BCVA between the Customized Setting 2 of DAVS Ver. 1.3 with 0.025% ICG and the Preset 3 of DAVS Ver. 1.1 with 0.075% ICG groups at baseline and at 12 months (*P* > 0.05, respectively).

**Conclusion:**

Customized DAVS settings enabled surgeons to use a 3-fold lower ICG concentration in ILM peeling.

## Introduction

Since Ekardt et al. (1) first reported internal limiting membrane (ILM) peeling in full-thickness macular hole (MH) surgery in 1997, this approach has been used routinely to improve MH closure rates (2, 3). In the epiretinal membrane (ERM) and MH, a meta-analysis reported that ILM peeling improves visual acuity in long-term follow-ups and decreases ERM recurrence rates (4). According to the preference and trends (PAT) survey of the American Society of Retina Specialists (ASRS), more than 60% of vitreoretinal surgeons in the United States and Europe are performing ILM peeling during ERM surgery (5). However, the translucency of the ILM causes significant difficulty in ILM peeling. Therefore, ILM staining methods have been developed, including indocyanine green (ICG), trypan blue (TB), and brilliant blue G (BBG) (6–8). Recently, ASRS conducted a PAT survey to determine whether adjuvant dyes and which ones are used to aid in macular surgery. The survey reported that 69.0% of surgeons in the United States are still using ICG; 47.5% of surgeons in Asia/Pacific are still using ICG, and 39.9% of surgeons in Europe are using ICG or TB (5). In addition, ICG selectively stains ILM, and it is superior to TB in terms of the staining intensity which could be advantageous in cases with strong vitreoretinal adhesion (9). However, several studies have reported potential toxic effects of the ICG (10–12) and BBG dyes (13–15) used during macular surgery. Thus, protocols that minimize the use and amounts of these dyes would be clinically desirable.

Until recently, imaging devices have been designed to improve ILM contrast using multi-color endoillumination probes with light-emitting diode light sources (16). However, recently developed digitally assisted vitreoretinal surgery (DAVS), NGENUITY^®^ 3D Visualization system (Alcon, Fort Worth, TX, USA) enables surgeons to customize the image color profiles of 3D high dynamic range (HDR) surgical images in real time (17). Although previous studies have addressed that different color channels are available in DAVS (17–19), no prior studies have quantitatively measured and compared color contrast in different color settings. Furthermore, the effect of different color settings under different ICG concentrations has not been evaluated.

The present study evaluated the color contrast ratio (CCR) of images captured during vitrectomy using different DAVS color settings. Furthermore, we determined if customizing color settings enabled surgeons to lower the ICG dye concentration as much as 3-fold in the ILM peeling process.

## Methods

### Study Design and Participants

This prospective comparative observational study with consecutive patients was performed in the Department of Ophthalmology at Kyungpook National University. The protocol was approved by the Institutional Review Board of the Kyungpook National University Hospital (KNUH 01-015) and was conducted in accordance with the tenets of the Declaration of Helsinki.

The study group consisted of all consecutive Korean patients that underwent combined phacoemulsification (Centurion Vision System, Alcon) and 25G vitrectomy (Constellation Vision System, Alcon) with ILM peeling for MH or ERM. Patients enrolled from November to December 2018 were assigned to the standard operating microscope (SOM) (OMPI Lumera 700; Carl Zeiss Meditec, Inc., Germany) group. Patients enrolled in January 2019 were assigned to the DAVS version 1.1 (Ver. 1.1) (NGENUITY^®^, Alcon Inc., Fort Worth, TX, USA) group, and those enrolled in August 2020 were assigned to the DAVS Ver. 1.3 group. The visualization system used for surgery was not selected based on the patients' status. All surgeries were conducted by a single operator (DP). Exclusion criteria were as follows: presence of spherical equivalent ≥6.0 diopters or axial length ≥26 mm, history of previous ocular surgery, ocular trauma, and corneal diseases, including corneal opacity or corneal dystrophy.

### Digitally Assisted Vitreoretinal Surgery Settings and ICG dye Concentration

The NGENUITY^®^ system included a 3D HDR surgical camera with a complementary metal oxide semiconductor image sensor, a 3D compact image processing unit, and an OLED 3D 4K ultra high-definition 55-inch flat panel display (resolution 3,840 × 2,160 pixels; mode number: OLED55E6P; LG ltd., Seoul, Republic of Korea). The 4K OLED display was placed about 1.2 m from the operator, and operators wore passive circular polarizing eyeglasses.

Table 1 shows ICG dye concentrations and parameters for DAVS Ver. 1.1, and DAVS Ver. 1.3 groups. The 0.075% ICG dye concentration was sufficient for ILM visualization under the SOM, while 0.025% ICG was not sufficient (Supplementary Figure 1). Thus, all patients in the SOM group received a 0.075% solution (0.75 mg/mL) of ICG (Diagnogreen^^®^^; Daiichi Sankyo Co., Tokyo, Japan) diluted with balanced salt solution (BSS, Alcon). In the DAVS Ver. 1.1 group, the same ICG dye concentration of 0.075% was used to determine the optimal DAVS system preset. However, in the DAVS Ver. 1.3 group, to determine if the customized settings of DAVS Ver. 1.3 enabled use of a lower ICG dye concentration, the ICG dye concentration was decreased to 0.025% (0.25 mg/mL). In all the cases, after the removal of the ERM, a volume of 0.05 mL of the diluted ICG was injected only once into the vitreous cavity to stain the ILM for 30 s and then quickly washed out. The stained ILM was removed using 25G ILM forceps (GRIESHABER REVOLUTION^®^ DSP ILM Forceps, Alcon).

**Table 1:** Concentrations of indocyanine green dye and parameters for digitally assisted vitreoretinal surgery groups.

	**DAVS Ver. 1.1**				**DAVS Ver. 1.3**	
**Variables**	**Default**	**Preset 1**	**Preset 2**	**Preset 3**	**Customized setting 1**	**Customized setting 2**
ICG dye concentration, %	0.075	0.075	0.075	0.075	0.025	0.025
Red, Green, Blue, %	100,100,100	20,100,100	80,80,100	85,100,90	86,100,100	90,100,100
Hue, °	+2	+2	+2	+2	+2	+20
Saturation, %	90	90	90	90	90	100
Gamma	1.2	1.2	1.2	1.2	1.2	0.9
Screen distance, meter	1.2	1.2	1.2	1.2	1.2	1.2
Aperture, %	50	50	50	50	50	50
Endoillumination power, %	35	35	35	35	35	35

*ICG, indocyanine green; DAVS, digitally assisted vitreoretinal surgery; Ver., version*.

In the DAVS Ver. 1.1 group, macular and vitreous images were captured four times continuously following four different color channels for each patient: Default [Red (R) 100, Green (G) 100, Blue (B) 100%]; Preset 1 (R 20, G 100, B 100%); Preset 2 (R 80, G 80, B 100%); and Preset 3 (R 85, G 100, B 90%). Hue, saturation, and gamma values were the same in all presets (+2°, 90%, and 1.2, respectively).

In the DAVS Ver. 1.3 group, macular images were captured twice continuously following two different customized settings for each patient: Customized Setting 1 (R 86, G 100, B 100%, Hue +2°, Saturation 90%, Gamma 1.2) and Customized Setting 2 (R 90, G 100, B 100%, Hue +20°, Saturation 100%, Gamma 0.9).

Other parameters, including endoillumination power, screen distance, and aperture, were identical in all groups. We consistently used plano contact lenses (Machemer Flat vitrectomy Lens OLV-5^®^, Ocular instruments, Inc., USA) when performing ILM peeling and located the endoilluminator in the mid-vitreous cavity in which the light pipe was not visible from the surgeon's visual field during the ILM peeling.

### Data Analysis and Ophthalmologic Examination

**Figure 1:**
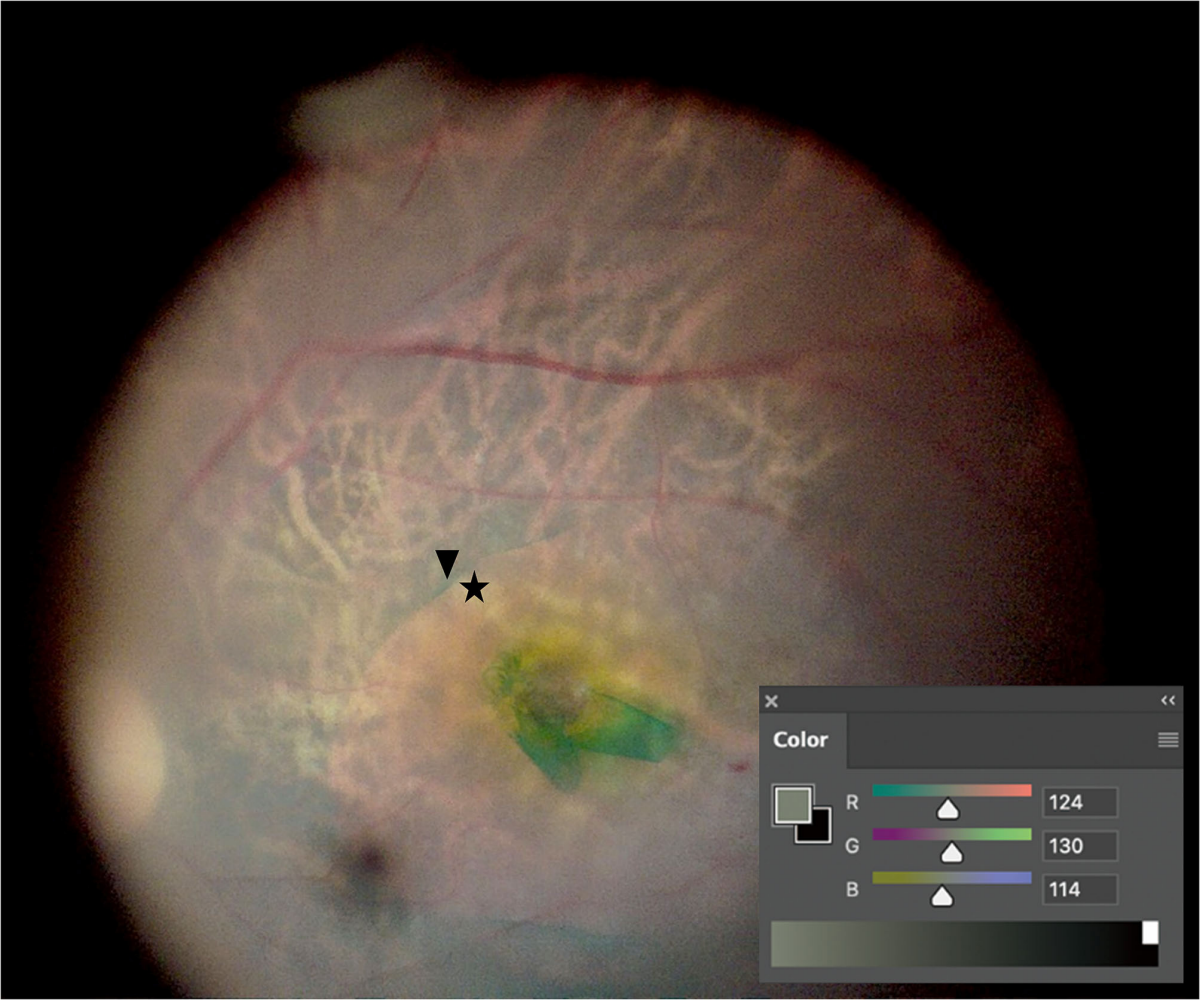
Color contrast ratio (CCR) measurement. After internal limiting membrane (ILM) peeling, retinal images were captured. CCR was measured by comparing RGB values between the indocyanine green-stained area (arrowhead) and the ILM-peeled area (asterisk) using the Eyedropper tool of Photoshop.

Vitrectomy and ILM peeling were conducted after phacoemulsification, and intraocular lens (IOL) implantation was subsequently conducted. Retinal images were captured after ILM peeling using a microsurgical operating camera (MediLive Trio Eye, Panasonic, Germany) (Figure 1). Because retinal images were captured in the aphakic state, neither cataract nor IOL color affected the contrast of the captured retinal images. The RGB values of the images were analyzed using the Eyedropper tool of Adobe Photoshop CC 20.0.8 software (Adobe System, San Jose, CA, USA). RGB values were analyzed in masked images by two retinal specialists (SP and JD). The interobserver agreement for RGB values was satisfactory (interclass correlation coefficient = 0.922, *P* < 0.001). Color luminance and CCR were calculated according to previous studies (20). In summary, If the *R* < 0.03928, Rs is estimated by using Rs = R/12.92. If the R value was more than 0.03928, Rs is estimated by using Rs = [(R + 0.055)/1.055]^2.4^ The Gs and Bs value can be estimated in the same manner. And then, the Color luminance and CCR are estimated by using the following: Color Luminance (L) = 0.2126Rs + 0.7152Gs + 0.0722Bs and CCR = (Lmax + 0.05)/(Lmin + 0.05), where Lmax = luminance of the brighter background and Lmin = luminance of the darker background. White balance was calibrated at the start of surgery.

All patients underwent full ophthalmologic examinations, including BCVA (logarithm of the minimal angle of resolution, logMAR), intraocular pressure (IOP) measurement, slit-lamp examination, and fundus examination at baseline and at every postoperative visit for at least for 12 months.

### Statistical Analyses

Statistical analyses were performed using SPSS v.18.0 for Windows (SPSS Inc., Chicago, IL, USA). Quantitative data are expressed as mean ± standard deviation, and qualitative data are expressed as percentages. Mann-Whitney *U*-test and Kruskal-Wallis test were used to compare quantitative data, while Chi-square tests were used to compare qualitative data. Comparison of CCR between different color channels was analyzed using a Kendall's *W*-test and Wilcoxon signed-rank test. Multiple comparisons were adjusted by the Bonferroni method. *P* < 0.05 were considered statistically significant.

Considering our previous pilot studies, the required sample size to detect a statistical significance of 5%, a power of 80%, and a drop rate of 10% was 12 eyes (12 patients) per group.

## Results

The study included 50 eyes (50 patients) with ERM (*n* = 45 eyes) or MH (*n* = 5 eyes). Table 2 shows patient clinical characteristics for the SOM (*n* = 25 eyes; 22 ERM and 3 MH), DAVS Ver. 1.1 (*n* = 12 eyes; 11 ERM and 1 MH), and DAVS Ver. 1.3 groups (*n* = 13 eyes; 12 ERM and 1 MH). Mean patient age was 68.3 ± 6.7 years (range, 61–82 years) in the SOM group, 69.3 ± 6.5 years (range, 60–83 years) in the DAVS Ver. 1.1 group, and 67.1 ± 6.2 (range, 61–80 years) in the DAVS Ver. 1.3 group, which did not differ between groups (*P* > 0.05). The groups did not differ in sex distribution, indication of surgery, baseline BCVA, IOP, or axial length (*P* > 0.05, respectively).

**Table 2:** Clinical characteristics of standard operating microscope and digitally assisted vitreoretinal surgery groups at baseline.

**Variables**	**SOM (*n* = 25)**	**DAVS Ver. 1.1 (*n* = 12)**	**DAVS Ver. 1.3 (*n* = 13)**	*P*-value^*^
Age, years	68.3 ± 6.7	69.3 ± 6.5	67.1 ± 6.2	0.749^†^
Male/female, *n*	10/15	7/5	3/10	0.199^‡^
Indication of surgery (ERM/MH)	22/3	11/1	12/1	0.656^‡^
BCVA, logMAR	0.76 ± 0.44	0.77 ± 0.61	0.54 ± 0.50	0.090^†^
IOP, mmHg	13.8 ± 2.7	15.0 ± 4.5	13.4 ± 2.7	0.708^†^
Axial length, mm	23.51 ± 0.81	23.85 ± 0.70	23.78 ± 1.19	0.204^†^

*Data are shown as the mean ± standard deviation unless indicated otherwise.*

*BCVA, best-corrected visual acuity; ICG, indocyanine green; logMAR, logarithm of the minimal angle of resolution; IOP, intraocular pressure; SOM, standard operating microscope; DAVS, digitally assisted vitreoretinal surgery; Ver., version; ERM, epiretinal membrane; MH, macular hole.*

* *P-values were compared between SOM, DAVS version 1.1 and DAVS version 1.3 groups.*

† *Kruskal-Wallis test.*

‡ *Chi-square test*.

### Measurement of Color Contrast Ratio in DAVS Ver. 1.1 With 0.075% ICG

**Figure 2:**
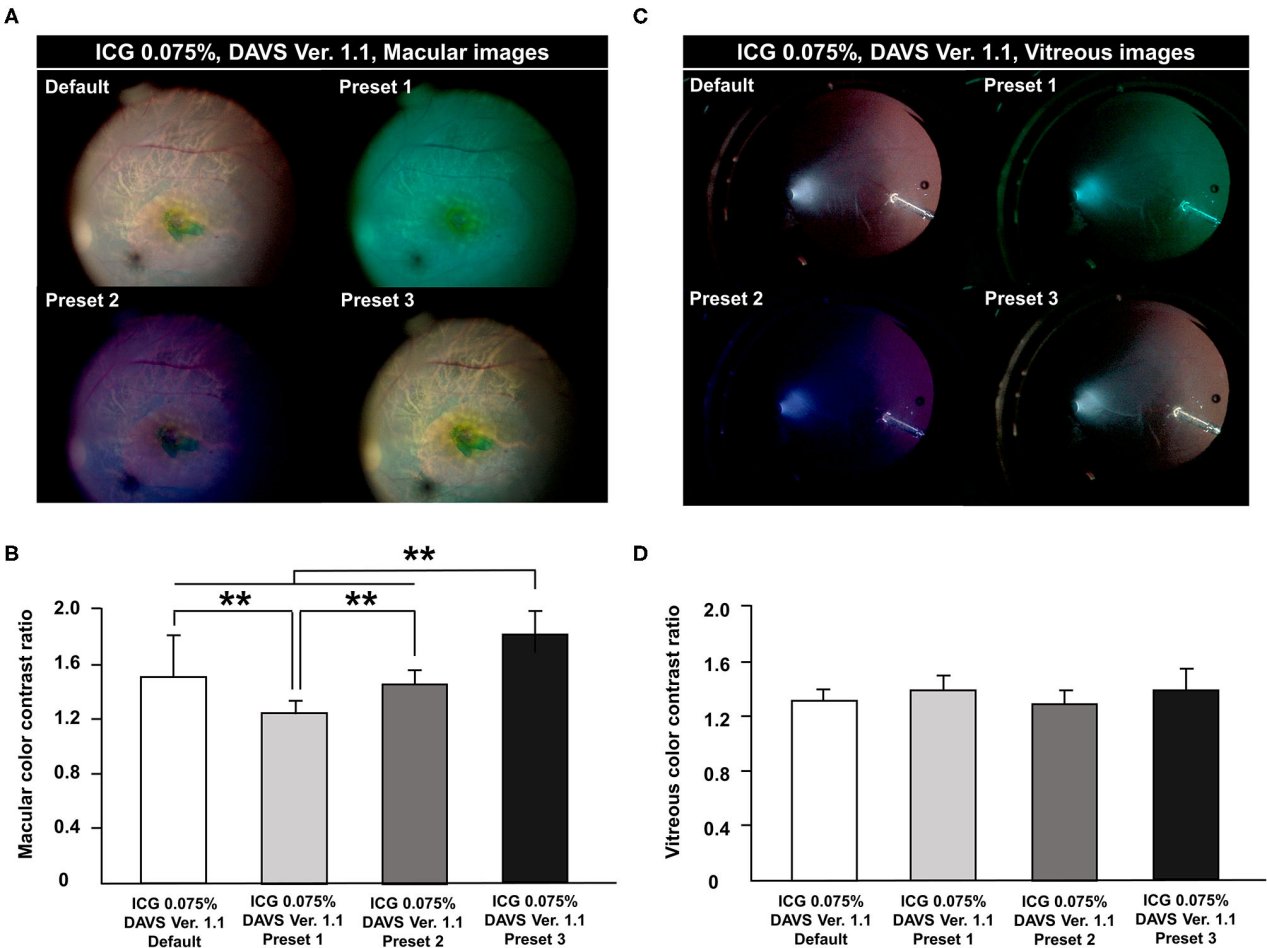
Comparison of color contrast ratio (CCR) among channels in digitally assisted vitreoretinal surgery system (DAVS) Ver. 1.1. CCR was compared in macular and vitreous images from the DAVS Ver. 1.1 group. The following four channels were compared in each captured macular and vitreous image: Default [Red (R) 100%, Green (G) 100%, and Blue (B) 100%]; Preset 1 (R 20, G 100, B 100%); Preset 2 (R 80, G 80, B 100%); and Preset 3 (R 85, G 100, B 90%). **(A,B)** Macular CCR was different among the four channels (*P* < 0.01). CCR was highest in Preset 3 and lowest in Preset 1 (*P* < 0.01). **(C,D)** In vitreous images, CCR did not differ among the four settings (*P* = 0.294). **P < 0.01.

During the first study period with DAVS Ver. 1.1, to determine the optimal color channels in the DAVS system, we compared the CCR among four different presets with 0.075% ICG dye. In macular images, the CCR differed among presets, with the highest CCR in Preset 3 and the lowest CCR in Preset 1 (Figures 2A,B, *P* < 0.01). However, in the vitreous images, CCR did not differ among presets (Figures 2C,D, *P* = 0.294).

### Measurement of Color Contrast Ratio in DAVS Ver. 1.3 With 0.025% ICG

**Figure 3:**
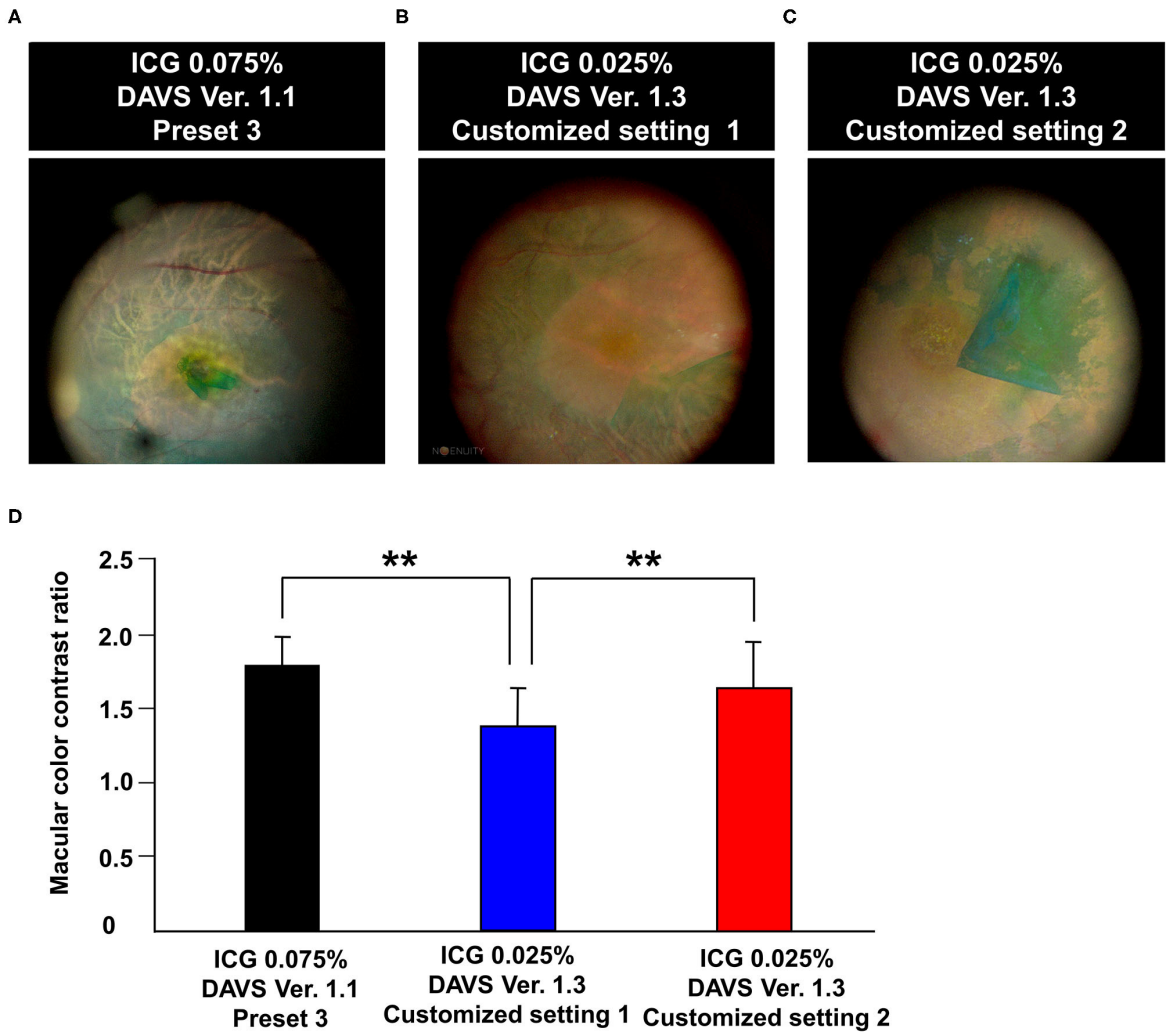
Comparison of color contrast ratio (CCR) using different color settings of digitally assisted vitreoretinal surgery system (DAVS) in different indocyanine green (ICG) concentrations. **(A)** Macular image captured using Preset 3 of DAVS Ver. 1.1 with 0.075% ICG. **(B,C)** Macular image captured using Customized Settings 1 and 2 of DAVS Ver. 1.3 with 0.025% ICG. **(D)** CCR of Customized Setting 2 of DAVS Ver. 1.3 with 0.025% ICG did not differ from that of Preset 3 of DAVS Ver. 1.1 with 0.075% ICG (*P* = 0.887). **P < 0.01.

During the second study period with DAVS Ver. 1.3, to determine if detailed customized settings enabled lowering of the ICG dye concentration, we used 0.025% ICG dye. As shown in Table 1, the parameters of Customized Setting 1 of DAVS Ver. 1.3 were similar to Preset 3 of DAVS Ver. 1.1. However, the macular CCR from Customized Setting 1 of DAVS Ver. 1.3 was lower than that of the Preset 3 of DAVS Ver. 1.1 (Figures 3A,B,D, *P* < 0.01).

Subsequently, we modified parameters of color settings, including hue values (+2° to +20°), saturation (90% to 100%), and gamma (1.2 to 0.9), which were assigned as Customized Setting 2. The CCR of Customized Setting 2 was higher than that of Customized Setting 1 (Figures 3B–D, *P* < 0.01). Furthermore, the CCR of Customized Setting 2 of DAVS Ver. 1.3 with 0.025% ICG did not differ from that of Preset 3 of DAVS Ver. 1.1 with 0.075% ICG (Figures 3A,C,D, *P* = 0.887).

### Comparison of Intraoperative Complication Rates and Postoperative Best-Corrected Visual Acuity

There was no difference in the ILM peeling time between the SOM with 0.075% ICG and Setting 2 of DAVS Ver. 1.3 groups with 0.025% ICG (3.3 ± 1.9 vs. 3.4 ± 0.8 min, *P* = 0.361). Regarding intraoperative complications, no iatrogenic retinal tears or retinal detachments occurred in any groups. Compared with baseline, the BCVA at 12 months was significantly improved: 0.33 ± 0.39 in the Customized Setting 2 of DAVS Ver. 1.3 with 0.025% ICG group and 0.36 ± 0.31 in the Preset 3 of DAVS Ver. 1.1 with 0.075% ICG group (*P* < 0.05, respectively). However, there was no significant difference in BCVA between the two groups at 12 months (*P* = 0.406). All of the MH cases from three groups showed MH closure at 12 months and did not require secondary surgery. BCVA at 12 months from the SOM group was 0.43 ± 0.05 logMAR, the BCVA from the DAVS Ver 1.1 with 0.075% ICG group was 0.40 logMAR, and the BCVA from the DAVS Ver 1.3 with 0.025% ICG group was 0.40 logMAR.

## Discussion

Since the advent of DAVS, this platform has been reported to provide a larger field of view at a higher magnification with accurate focus, better educational value, and a more ergonomically comfortable position for the operator than the SOM system (17, 18, 21–24). Importantly, Gonzalez-Saldivar and Chow (25) reported that DAVS provided superior depth of field and lateral resolution than did SOM when the aperture and display distance settings of DAVS were optimized. Although several prior reports mentioned about the color channel function of DAVS (17–19), no previous studies have quantitatively measured macular color contrast according to different color channels or determined whether customized settings are able to lower ICG concentration.

ICG dye is commonly used for ILM visualization in macular surgery, and most surgeons use an ICG concentration of 0.1–0.5% (18, 26). However, several studies have reported the potential for ICG toxicity (Supplementary Table 1) (11, 27–30). Brief exposure of cultured human retinal pigment epithelial (RPE) cells to 0.1% decreases mitochondrial enzyme activity, suggesting the potential for toxicity (10). In macular surgeries, in 45% of cases, RPE atrophy occurred under 0.1% ICG at the site of a previous MH, which was confirmed by fundus photography (11), and RPE pigmentary changes were observed in 27% of patients with 0.25% ICG (31).

Despite its potential toxic effects, ICG is widely used for ILM staining in clinic. According to a previous meta-analysis based on comparative studies published between 2004 and 2014, 82.4% of studies used ICG. The study demonstrate that ICG is still one of the primary adjuvants used clinically for ILM peeling. In a previous study, comparing electrophysiologic and histological findings after injecting different concentrations of ICG (0.05, 0.5, and 2.5%) into the vitreous cavity of rabbits, ICG had a dose-dependent toxic effect, as demonstrated by decreased retinal function and morphology consistent with toxicity (32). Thus, it is important for retinal surgeons to use as little ICG as possible.

However, under the microscope, ILM visualization is difficult at lower concentrations such as 0.025% (Supplementary Figure 1). Furthermore, Kwok et al. reported that 50% of cases with 0.025% ICG required second ILM staining with 0.125% ICG due to poor ILM visualization (33). Thus, we sought to determine whether customized color settings could maximize visualization of the ICG-stained ILM, allowing the use of lower ICG concentrations.

During the first study period with DAVS Ver. 1.1, we determined which color channels allowed maximal color contrast in 0.075% ICG-stained ILM. Preset 3 had the highest CCR of the four evaluated color channels, likely because Preset 3 decreased the red and blue channels, emphasizing the green channel. This result suggested that green-emphasizing color channels are useful for visualization of the ICG-stained ILM.

Some reports have suggested that the blue channel increases visibility of the vitreous (17), which was not corroborated by the present study. Because the vitreous body is a transparent material and has no background, significant contrast would be difficult to visualize. However, it is possible that vitreous contrast varies depending on parameters of the surgical environment, such as the illuminator angle and location of the illuminator in the vitreous cavity.

Considering that the highest CCR was in Preset 3 of DAVS Ver. 1.1, we expected that using an ICG concentration lower than 0.075% for ILM visualization would be possible with the DAVS system. Thus, during the second study period with DAVS Ver. 1.3, we used 0.025% ICG and measured macular CCR. First, we used Customized Setting 1 of DAVS Ver. 1.3 similar to Preset 3 of DAVS Ver. 1.1, and subsequently compared the CCR between 0.025 and 0.075% ICG concentrations. The macular CCR from Customized Setting 1 of DAVS Ver. 1.3 was lower than that of Preset 3 of DAVS Ver. 1.1, likely due to the 3-fold decrease of ICG concentration. The above results suggest that adjustment of RGB channels alone was insufficient to visualize the ILM at 0.025% ICG. Rather, it was necessary to also adjust other parameters, such as hue, saturation, and gamma values. Thus, we modified the customized setting using other parameters.

Since the development of DAVS Ver. 1.2, customized settings of anterior, macular, and posterior image modes have been made available to optimize viewing experience during surgical procedural steps. Furthermore, not only RGB, but also hue, saturation, and gamma values can be easily modified to create customized settings for each surgical situation.

Various parameters related to color settings determine color perception including hue, saturation, and gamma values. Hue is used to distinguish colors, which is specified as between 0 and 360°. Saturation refers to the color intensity, with a higher saturation having more vivid color. Gamma is also important in digital image systems. While a high gamma setting can compress bright areas and increase black areas to create crisp and high-contrast images, a low gamma setting enables more detailed visualization in bright areas while still compressing shadow (34). Following the above principle, in Customized Setting 2, hue and saturation values were increased and gamma value was decreased to improve macular color contrast at 0.025% ICG. Interestingly, the present study demonstrated that Customized Setting 2 of DAVS Ver. 1.3 with 0.025% ICG exhibited a similar CCR to that of Preset 3 of DAVS Ver. 1.1 with 0.075% ICG. A recent questionnaire survey reported similar results that higher Hue parameter was correlated with better visualization (19). This suggested that the customized color settings available in the DAVS system enabled surgeons to lower the ICG concentration as much as 3-fold, which would be helpful for reduction of ICG toxicity.

This study has several limitations. First, the sample size was small in the DAVS groups. However, the sample size was sufficient for analysis by sample size calculation. Second, we could not directly compare CCR between the SOM and DAVS groups because the digital image resolution was not the same between the two systems. Third, because BBG and TB are not commercially available in Korea, we were unable to measure CCR using these dyes. Several cases have reported retinal toxicity resulting from the use of BBG dye during macular surgery (13–15). Although some studies have suggested a good safety profile for use of TB in macular surgeries (35, 36), a case study in which retinal toxicity occurred due to prolonged TB exposure has also been reported (37). Thus, it would be valuable to optimize instrument settings to maximize CCR during surgeries performed using BBG and TB to decrease the necessary dye concentration for ILM visualization, which we will evaluate in a future study. Fourth, although the same endoillumination power of 35% was used in the SOM and DAVS groups, this power is higher than is typically used for DAVS (24). In a future study, we will verify and optimize the settings using a lower endoillumination power. Fifth, although high myopic patients were excluded to minimize the effect of myopia on CCR, variations in myopic state and RPE pigmentation between patients could affect the macular CCR. In the future, it will be necessary to evaluate the correlation between the pigmentation state and baseline color conditions. Sixth, though the DAVS Ver. 1.1 group was allocated to find the optimal settings among the various presets and to compare with the DAVS Ver. 1.3, the DAVS Ver. 1.1 is no longer commercially available in the real-world DAVS. However, the approach used in this study to determine the optimized customized settings could be helpful for surgeons to adjust to the next versions of the DAVS system in the future. Seventh, due to the small proportion of MH cases, postoperative toxicity assessment such as fundus autofluorescence was not evaluated. However, the authors would like to say that the purpose of this study was to determine the optimal color settings to lower the ICG concentration because BBG and TB are not commercially available in Korea. In the future, we will evaluate how the reduced ICG concentration enabled by the optimized use of the DAVS system could affect the RPE toxicity. Eight, we could not measure the direct correlation between the CCR and the surgeons' subjective visual perception. Instead, the ILM peeling time between the SOM with 0.075% ICG and Customized Setting 2 with 0.025% ICG groups was compared which was not statistically different. The above data could suggest that the customized settings of the DAVS determined by trial and error to acquire a higher CCR could be meaningful for the surgeons' visual perception.

In conclusion, this is the first report comparing the color contrast between different DAVS settings and different ICG concentrations. The study demonstrated that the customized color settings of the DAVS system enabled surgeons to lower ICG concentration used, which would be advantageous in macular surgery.

## Data Availability Statement

The data that support the findings of this study are available from the corresponding authors upon reasonable request.

## Ethics Statement

The studies involving human participants were reviewed and approved by Institutional Review Board of Kyungpook National University Hospital (KNUH 01-015). The patients/participants provided their written informed consent to participate in this study.

## Author Contributions

SP, JD, JS, and DP contributed to the conception or design of the work. SP, JD, and DP contributed to the data collection, data analysis and/or interpretation. All authors contributed to the drafting of the article and critical review of the article.

## Funding

This work was supported by Biomedical Research Institute grant, Kyungpook National University Hospital (2018). This research was supported by a grant of the Korea Health Technology R&D Project through the Korea Health Industry Development Institute (KHIDI), funded by the Ministry of Health and Welfare, Republic of Korea (Grant number: HI15C0001).

## Conflict of Interest

The authors declare that the research was conducted in the absence of any commercial or financial relationships that could be construed as a potential conflict of interest.

## Publisher's Note

All claims expressed in this article are solely those of the authors and do not necessarily represent those of their affiliated organizations, or those of the publisher, the editors and the reviewers. Any product that may be evaluated in this article, or claim that may be made by its manufacturer, is not guaranteed or endorsed by the publisher.
