# Corrigendum: Customized Color Settings of Digitally Assisted Vitreoretinal Surgery to Enable Use of Lower Dye Concentrations During Macular Surgery

**DOI:** 10.3389/fmed.2022.901546

**Published:** 2022-04-21

**Authors:** Su Jin Park, Jae Rock Do, Jae Pil Shin, Dong Ho Park

**Affiliations:** ^1^Department of Ophthalmology, School of Medicine, Kyungpook National University, Kyungpook National University Hospital, Daegu, South Korea; ^2^Kyungpook National University Bio-Medical Research Institute, Daegu, South Korea; ^3^Kyungpook National University Cell and Matrix Research Institute, Daegu, South Korea

**Keywords:** vitrectomy, macular surgery, epiretinal membrane (ERM), ILM peeling, 25 gauge pars plana vitrectomy

In the original article, we neglected to include the funder “Korea Health Industry Development Institute (KHIDI), Grant number: HI15C0001” in the Funding section. It should be “This work was supported by Biomedical Research Institute grant, Kyungpook National University Hospital (2018). This research was supported by a grant of the Korea Health Technology R&D Project through the Korea Health Industry Development Institute (KHIDI), funded by the Ministry of Health and Welfare, Republic of Korea (Grant number: HI15C0001).”

In the published article, there was an error in affiliation 1. Instead of “Department of Ophthalmology, School of Medicine, Kyungpook National University Hospital, Kyungpook National University, Daegu, South Korea,” it should be “Department of Ophthalmology, School of Medicine, Kyungpook National University, Kyungpook National University Hospital, Daegu, South Korea.”

The authors apologize for these errors and state that this does not change the scientific conclusions of the article in any way. The original article has been updated.

## Publisher's Note

All claims expressed in this article are solely those of the authors and do not necessarily represent those of their affiliated organizations, or those of the publisher, the editors and the reviewers. Any product that may be evaluated in this article, or claim that may be made by its manufacturer, is not guaranteed or endorsed by the publisher.

