# Supplementary material for: Customized Color Settings of Digitally Assisted Vitreoretinal Surgery to Enable Use of Lower Dye Concentrations During Macular Surgery
Source: Front Med (Lausanne). 2022 Jan 24;8:810070. doi: 10.3389/fmed.2021.810070 (PMC8818890; doi:10.3389/fmed.2021.810070)
Supplement: Supplementary file 1 [file Table_1.DOC]

Supplementary Material

# Customized Color Settings of Digitally Assisted Vitreoretinal Surgery to Enable Use of Lower Dye Concentrations during Macular Surgery

**Su Jin Park^1^, Jae Rock Do^1^, Jae Pil Shin^1^, Dong Ho Park^1,2*^**

*** Correspondence:**Dong Ho Park, MD, PhD
DongHo_Park@knu.ac.kr

**
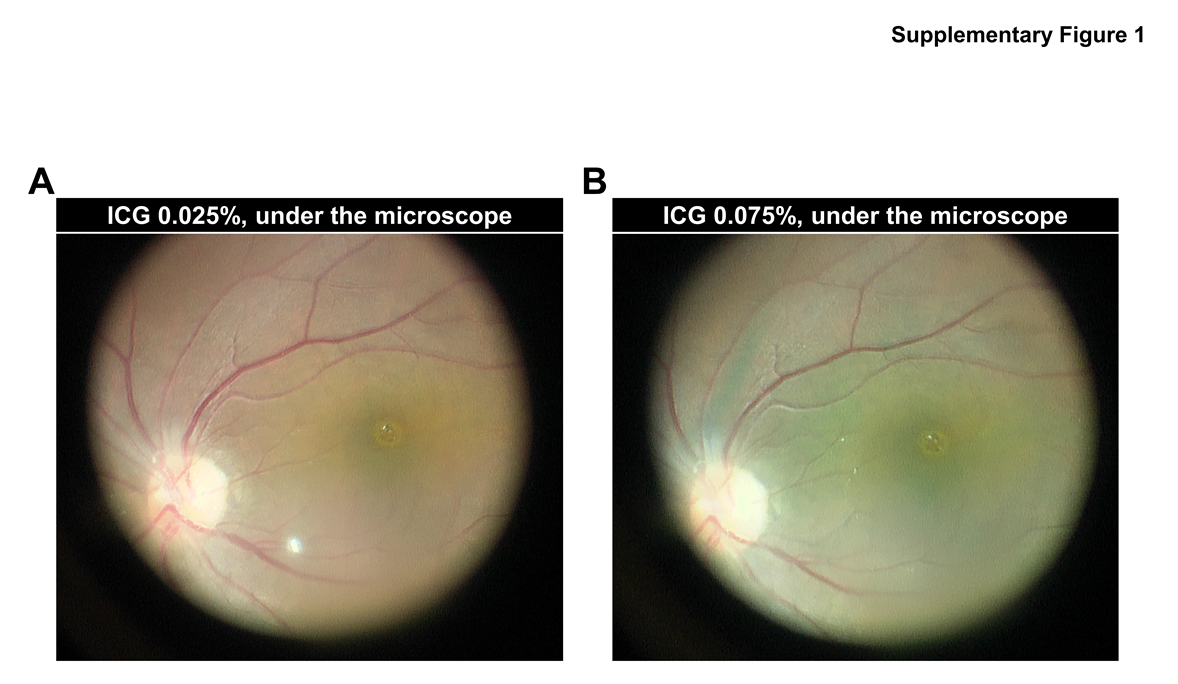
**

**Supplementary Figure 1.** Macular images captured using **(A)** 0.025% ICG and **(B)** 0.075% ICG in a standard operating microscope.

**Supplementary Table 1. Postoperative complications according to various concentrations of indocyanine green**

| **Study** | **Indocyanine green** | | | **Postoperative complications** |
| --- | --- | --- | --- | --- |
|  | **Concentration (%)** | **Volume  (mL)** | **Exposure time (minute)** |  |
| Haritoglou et al., 2003 | 0.05 | Up to 0.5 | 1 | 35% visual field defect |
| Engelbrecht et al., 2002 | 0.1 | 1-2 | 0.5–2 | 54.5% RPE changes |
| Cheng et al., 2005 | 0.25 | 1.5 | 2 | RPE atrophy, optic nerve atrophy |
| Uemoto et al., 2005 | 0.5 | NA | 1 | 9.7% RPE changes |
| Posselt et al., 2005 | 0.5 | 0.2–0.4 | 1–3 | 50% RPE changes |

NA, data not available
